# Household Power Demand Prediction Using Evolutionary Ensemble Neural Network Pool with Multiple Network Structures

**DOI:** 10.3390/s19030721

**Published:** 2019-02-10

**Authors:** Songpu Ai, Antorweep Chakravorty, Chunming Rong

**Affiliations:** Department of Electrical Engineering and Computer Science, University of Stavanger, 4036 Stavanger, Norway; antorweep.chakravorty@uis.no (A.C.); chunming.rong@uis.no (C.R.)

**Keywords:** machine learning, artificial neural network, smart sensor, evolutionary algorithm, ensemble learning, long short-term memory, gated recurrent unit, demand prediction, HEMS, missing data

## Abstract

The progress of technology on energy and IoT fields has led to an increasingly complicated electric environment in low-voltage local microgrid, along with the extensions of electric vehicle, micro-generation, and local storage. It is required to establish a home energy management system (HEMS) to efficiently integrate and manage household energy micro-generation, consumption and storage, in order to realize decentralized local energy systems at the community level. Domestic power demand prediction is of great importance for establishing HEMS on realizing load balancing as well as other smart energy solutions with the support of IoT techniques. Artificial neural networks with various network types (e.g., DNN, LSTM/GRU based RNN) and other configurations are widely utilized on energy predictions. However, the selection of network configuration for each research is generally a case by case study achieved through empirical or enumerative approaches. Moreover, the commonly utilized network initialization methods assign parameter values based on random numbers, which cause diversity on model performance, including learning efficiency, forecast accuracy, etc. In this paper, an evolutionary ensemble neural network pool (EENNP) method is proposed to achieve a population of well-performing networks with proper combinations of configuration and initialization automatically. In the experimental study, power demand predictions of multiple households are explored in three application scenarios: optimizing potential network configuration set, forecasting single household power demand, and refilling missing data. The impacts of evolutionary parameters on model performance are investigated. The experimental results illustrate that the proposed method achieves better solutions on the considered scenarios. The optimized potential network configuration set using EENNP achieves a similar result to manual optimization. The results of household demand prediction and missing data refilling perform better than the naïve and simple predictors.

## 1. Introduction

With the progress of technology in the energy field, electricity can be generated, transported and stored more conveniently, efficiently and economically [1]. This tendency inspires further applications with the acceleration of Internet of Things (IoTs) in smart grids [2], which leads to an increasingly complicated electric environment in a smart city. As an essential portion of the smart grid, the low-voltage local microgrid faces a qualitative change with the popularization of electric vehicle (EV) together with the upcoming micro-generation (e.g., solar and wind power micro-generation) and local storage products.

The existing electricity infrastructure lacks mechanisms to handle mass and concentrated energy demands primarily led by EV charging requirements. At the same time, current simplex supply-demand relationship in the market between the producer and customer is no longer accomplished to support the role of prosumer (for instance, a household with micro-generation and local storage) in the microgrid. It becomes necessary to develop a home energy management system (HEMS) to efficiently integrate and manage household energy micro-generation, consumption and storage, in order to realize decentralized local energy systems at the community level.

Predicting domestic demand is of great importance for establishing HEMS. It can be utilized to reduce the damage caused by power overload and to realize load balancing at neighborhood and community level. Demand prediction of a household is also required by the solution of reducing potential demand peaks from outer grid by pausing the operation of controllable power intensive non-critical loads or by utilizing real-time generated renewable power and locally stored power within the household network whenever a high demanding period is forecasted. Furthermore, precise predictions on household demand are additionally required by other local smart energy solutions including vehicle-to-grid system, micro-generation and storage system, neighborhood energy sharing, etc. Therefore, domestic demand prediction is indispensable for constructing HEMS. 

With the support of IoT techniques such as smart meters and wireless sensor network, much more adequate and detailed power usage data are available to be processed and analyzed using AI to realize HEMS.

Artificial neural network (ANN), as a promising prediction method, has emerged in both academia and industry for years. ANNs model biological networks in the brain to achieve machine learning. ANNs with multiple hidden layers between the input and output layers are also called deep neural networks (DNNs). Numerous existing papers on energy prediction in power grid are based on ANNs and DNNs with specific network configurations particularly designed for the study cases [3,4,5,6,7]. The terminology “configuration” in this paper indicates the arrangement of the mentioned network’s functional units, including the type of the network, the specific structure of the network, the parameters of the neurons/units, etc.

In addition, an extended network structure, recurrent neural network (RNN) is utilized in the demand prediction, which is presented to be able to keep information within its internal state (memory) [8]. Moreover, more complicated recurrent units, such as long short-term memory (LSTM) unit [9] and gated recurrent unit (GRU) [10], are developed as individual components of a network for RNNs to promote the advantage of memory by gated state. One or multi-layers of LSTM units and GRUs is argued to have the capability of learning lags with different time scales [11]. The potential network types and structure sets are normally selected in advance in the existing studies adopted LSTM and GRU based RNNs [12,13,14,15].

The selection of network configuration for each research is generally a case by case study. The proposed network configurations are normally decided through empirical or enumerative approaches. Moreover, the commonly utilized initialization method also provides uncertainty to the model performance. The weights and biases in the network are usually assigned based on random numbers. This causes diversity on model performance, including learning efficiency, forecast accuracy, etc. 

These characteristics of using neural network-based methods have led researchers to provide manual tuning for each specific case to discover a suitable network configuration with a proper initialization. There is a lack of methodology to achieve an appropriate combination of network configuration and initialization for a given problem automatically. This type of method is required by the implantation of HEMS since it is not feasible to provide manual tuning for each household. The power demand of households could be very different based on diversities on location, number of people living inside, their ages, habits, etc. Hence the preferred network configuration of one household could be hard to share with another household. Although a potential network configuration set could be obtained by general analysis, an appropriate configuration and initialization combination is required to be supplied to each household specifically.

In this paper, an evolutionary ensemble neural network pool (EENNP) method is proposed to forecast household power demand. The main contributions of this paper are as follow:

- Propose the EENNP method to achieve a population of well-performing networks with proper combinations of configuration and initialization automatically.
- Investigate the performance of EENNP on household power demand prediction, with the discussion on the impacts of evolutionary parameters on the model performance.
- Explore the utilization of EENNP on optimizing the potential network configuration set of the method, comparing with manually optimizing process.
- Develop a missing data refilling method based on EENNP for the use of (near) real-time analyses.

The remainder of this paper is organized as follows. In Section 2, the ANN structures employed in the research and related works are presented. The proposed EENNP method is illustrated in Section 3. Afterwards, investigated smart sensor data and data pre-processing for the experimental study are described in Section 4. Section 5 demonstrates the performance analyses and discussions of the utilization of EENNP on the three application scenarios, before we conclude the paper in Section 6.

## 2. ANNs in Demand Prediction

In this section, the ANNs we employed in this paper are presented. Existing studies about predictions in energy field using different types of ANNs are introduced. 

ANN as a promising prediction method to handle unspecified non-linear relationship has emerged for years [16]. It models biological neural network as an interconnected group of nodes called neurons. In each neuron, weighted summation of an input vector is sent into the activation function to attain the output of the neuron. When an input is passed through the network by edges between neurons, a network output is attained. ANNs conduct learning through updating neurons’ weights after each training iteration. 

### 2.1. ANNs and DNNs

Conventionally, the term ANN also represents a specific type of neural network, which is a feedforward network with a single hidden layer. ANNs with multiple hidden layers are also called DNNs. More details on basic network principles of ANN and DNN can be found in [17,18].

ANNs and DNNs are widely utilized in smart home solutions to analyze sensor data. For instance, DNN is adopted by the authors of [3] to estimate human behaviors using insole sensor data. In [19], human activities in smart home are recognized using ANN. ANNs and DNNs are employed to discuss power profile recognition of electrical appliances in residential building [5]. On the direction of energy forecasting, ANNs and DNNs are involved in the prediction of the demand and consumption. An hourly energy consumption prediction of a university is practiced in [6] using ANN. In [20], an ANN based ensemble method is proposed to realize the hourly load forecasting on office buildings. ANN is adopted in [4] to forecast the hourly consumption of a company. In our previous work [7], the prediction of hourly consumption of a domestic household is investigated using ANN. However, among the existing studies, the configurations of the advised networks, such as the number of layers in the network, number of neurons in each layer and activation function of neurons, are generally selected through empirical decisions or enumerative experiments.

In order to design a method to obtain an efficient demand prediction for each household, whether the configuration of a network is appropriate for a specific household is not the focus of our study. A method suits more households or is capable to adjust itself for each household is then considered as economical. Thus, ensemble learning is chosen as a portion of the proposed method to reduce the risk of single ANN suddenly not working well. It is further discussed in Section 3.2. In addition, evolutionary method is adopted to make the ensemble more efficient. In fact, it is not necessary to well train all the ANNs to select out relatively more suitable individuals. More details are given in Section 3.1.

In fact, genetic algorithm, as a class of evolutionary method has been considered in existing studies as a method to optimize the performance of ANN based models. For instance, in [21], genetic algorithm is utilized in the solution to find the best schedule of energy appliances to achieve the desired energy reduction. However, genetic algorithm in this work is utilized to optimize the schedule of operation statuses of considered energy appliances, which is hard to be used on selecting better fitting ANNs in demand prediction. Moreover, in other genetic algorithm-based ANN optimizations such as in [22,23], etc., evolutionary methods are considered to be utilized on selecting edges between neurons or adjusting weights of one specific network structures. These utilizations are not appropriate for our study which seeks to select fitting individuals from a population of networks.

### 2.2. LSTM and GRU based RNNs

RNN is a further extended type of ANN, which contains one or multiple directed cyclic architectures to remain certain portion of information achieved from input data within the network to provide “memory” on learning and analyzing [24]. The directed cyclic architecture, called as unit in this paper, is the critical part of an RNN to “remember” the repeating behavior habits through time. However, depending on different unit designs, vanishing or exploding gradient problem could occur during the training process [25], which may hurt the capability of units to capture long-term dependency. 

**Figure 1:**
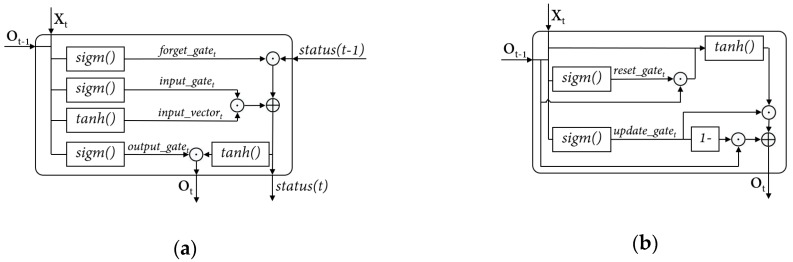
The architecture of special units in RNN: (**a**) a LSTM unit; (**b**) a GRU.

Complex recurrent units, such as LSTM [9] and GRU [10] are proposed to overcome the problem. These two types of units consist of several gate mechanisms interacting with each other to keep the information flow inside the unit [8]. The architectures of LSTM unit and GRU are illustrated in Figure 1.

Briefly, LSTM unit is considered as a cell with status and three gates. Status is the “memory” the cell keeps, which is adjusted among the training process assisted by the gates. The three gates are: the forget gate, input gate and output gate, which are utilized to “forget” (diluting unnecessary information in the status), “remember” (adding new information into the status), and provide output for the unit respectively. 

A GRU holds a simpler architecture than LSTM unit. As observed in Figure 1, two intuitive advantages of GRU over LSTM are the missing of cell status and the reducing of gate number, which reduces the required amount of computation. Additional details on the mechanisms of the two special units can be achieved at [10,24].

LSTM and GRU based (one- or multiple-layer) RNNs are adopted to forecast sensor data. In [12], LSTM based RNN is used to forecast ship behaviors with automatic identification system sensor data. Multiple LSTM networks with different structures are tested in [13] to forecast power demand of an individual building. Several commonly selected LSTM and GRU based RNN structures are tested in [15] to predict power consumption. In [14], GRU based RNNs are adopted to forecast household power demand on one second resolution.

Similar as ANN and DNN based studies, it is able to observe from the existing RNN studies that the preferred network structures are generally developed for each case specifically. In some studies, multiple networks structures are tested in order to explore a proper network structure using enumeration. 

Moreover, the network initializations in the existing ANN, DNN and RNN based predictive studies generally use methods based on random values. The weight vectors, biases in the networks are chosen at random within/without a range/distribution. For instance, a current commonly utilized initialization method is Xavier initialization [26], which bases on customized uniform distribution for each layer. The dependence of initialization method on random number causes uncertainties on the model training and forecast performance. As an example, different learning efficiency and forecast accuracy could be observed among a population of LSTM based RNNs with same network configuration but different initializations [27].

Within the implementation of HEMS, it is a challenge to obtain a population of appropriate combinations of network configuration and initialization for each household. The EENNP method introduced in the next section is developed to attempt to fill this gap.

## 3. Evolutionary Ensemble Neural Network Pool

**Figure 2:**
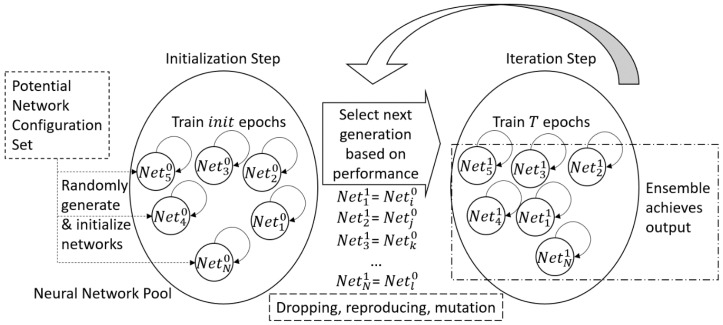
An overview of the proposed method.

In this section, the proposed EENNP method is presented to overcome the lacks discussed above. As shown in Figure 2, “neural network pool” refers to a population of neural networks existing in certain learning environment. It can be imaged as a crowd of fish living in a water pool in nature. Neural networks with different types can be seen as various species of fishes living in the pool. Networks with the same type but different structures and initializations can be considered as individuals of the same species. The learning environment is the “water pool” of the “fishes”. The pool every time gets some food (data) from the outer environment to feed all the fishes. The individuals who can adapt to the food are able to survive and reproduce, while the ones cannot learn the environment properly are dropped from the pool. Through generations of selections, the survived neural networks are the individuals and species fit to learn the environment. Hence, an ensemble output is provided by the survival networks in the pool jointly. This method could be suitable for the application scenarios such as discovering the potential network configuration set for a certain problem, determining the most appropriate one/subset from a set of configurations which is suitable for a specific case study, etc.

The details of the evolutionary process as well as the ensemble output of the neural network pool are proposed in the following subsections.

### 3.1. Evolutionary Process of EENNP

Evolutionary method is a type of global optimization algorithm which simulates biological evolution process existing in the natural environment. A population of potential problem solutions are selected through trial and error approach to realize optimization. Evolutionary methods with advantages on simplicity of conceptual design, feasibility on parallelism process, robustness in dynamic changes, capability for self-optimization [28], have been widely utilized to produce optimized solutions in wide ranges. Considering our objective of selecting potential network configuration set for household demand prediction as well as picking out better performing network configuration and initialization combinations to provide demand forecast for each specific household, it is interesting to adopt an evolutionary method to deal with.

Generally, the implementation procedure of an evolutionary method can be considered with two steps. The first step is to generate an initial population with stochastic candidate solutions for the problem. The second step is to iteratively update the population. This step is normally with three operations, which are evaluating all individuals, selecting best-fit individuals to reproduce with mutation, and replacing the least-fit population with the new individuals generated by reproduction. As a result, the fitness of the population for the problem is gradually evolved through training.

Specifically, the two steps in the proposed method are constructed as follows:

• Initialization Step:

A set of potential network configurations including network types, structures, activation functions for the problem is pre-decided based on a former research. Then, a population of networks are established in the pool. The configuration of each network is randomly picked from the potential set. Next, the networks are initialized by random numbers. In this paper, the number of networks in the pool is presented as N. A network individual i in the pool at iteration t is expressed as $Net_{i}^{t}$, where i∈[1,N]. For networks at the initialization step, t=0 is adopted.

Afterwards, all networks in the pool are trained by the same initial-training dataset for init epochs in order to indicate their suitability for the learning case.

• Iteration Step:

Except for the first iteration step just after the initialization step, all the survival networks in the pool should be trained by the training dataset for T epochs in the iteration step to advance their learning progress, in order to further evaluate their fitness.

The evaluation operation is executed after training. The performance of the networks is evaluated by a unique criterion using a set of untouched evaluation data. The networks are ranked by their fitness from best-fit to least-fit. The used evaluation dataset is then added into the training set for the training of the subsequent iteration steps.

The best-fit individuals are selected to reproduce themselves in the pool, while the least-fit individuals are removed from the pool to keep the population. In this paper, the percentage of best-fit networks to reproduce is denoted as $P_{select}$. All individuals remaining in the pool constitute the population of the next generation.

A mutation operation is executed here to add certain uncertainty into the reproduction. Each least-fit individual in the eliminating list gets a chance to survive to the next generation with a mutation possibility, which is expressed as $P_{mutation}$. Accordingly, the last few individuals in the best-fit reproducing list would not be able to reproduce in this iteration to keep the population of the pool.

Moreover, early-stopping is a commonly adopted method to avoid model overfitting through the iterative training process [29]. An early-stopping is triggered in the training of a model when the performance of the validation set stops improving by a number of epochs. Then the training process of that model is stopped before reaching the configured training epoch limit to avoid overfitting.

By training networks with different configurations and initializations, it could happen that a network individual in the pool achieves certain accuracy and stops improving, while other networks keep improving. If we keep training the stopped improving individual, it could lead over-fitting happening. This may cause the performance of the over-fitted network getting worse in evaluation and in the subsequent iterations. Therefore, early-stopping is adopted in the proposed method.

Whenever an early-stopping is triggered on an individual, the training process of that individual in current iteration step is finished immediately. Further operations such as evaluation, reproduction, etc., are executed when the training process of all the networks in the pool finish. Due to the training dataset is updated after each iteration step, all the networks need to be trained in the next iteration step even if an early-stopping is triggered in the current step.

Finally, after generations of iteration, a set of well-fitting networks with their configurations and initializations are selected out specifically suitable for the problem. These networks are then adopted together as a prediction model of the problem. The method which they collaboratively provide forecast output is demonstrated in the next subsection.

### 3.2. Ensemble Learning in EENNP

Ensemble learning is a type of machine learning which provides its output based on the combined outputs of multiple learning models [30]. The output of each model in an ensemble model can be seen as a candidate of the output. Through certain “election process”, an integrated result is obtained as the output of the ensemble model. Even though the integrated output is calculated by the candidates, it is not necessary that the output belongs to the candidate set. For instance, in [31], ensemble learning is adopted to forecast time series data using weighted mean of candidate models. In our previous work [32], a two-layer stacking ensemble model is developed to forecast the charging activity of electric vehicles. In [33], a neural network-based ensemble method is proposed, where ensemble is adopted to train a set of manual selected neural networks, each with better performance on certain portion of the forecast to obtain better results.

In this paper, the output of the pool is calculated by an ensemble method. Ensemble method is adopted here to reduce the risk that one network suddenly not working well by averaging the outputs from a population of networks with various configurations and initializations. The outputs of individuals in the pool are the candidates of the ensemble method. The word “pool” in the EENNP can also be seen with a similar implication as the “pool” within the pooling layer in the convolutional neural networks, where pooling is a form of non-linear down-sampling, combining the outputs of a population of neurons to provide an integrated output to represent the state of the population. The ensemble method in EENNP is utilized to capture the dynamic trade-off between networks with different configurations and initializations.

In the case study, average pooling is adopted to obtain the integrated output, as shown in (1).

(1) $$Out^{t}=\frac{1}{N}\sum_{i}^{N}Out_{Net_{i}^{t}},$$

where $Out^{t}$ represents the output of the network pool at step *t*.

**Figure 3:**
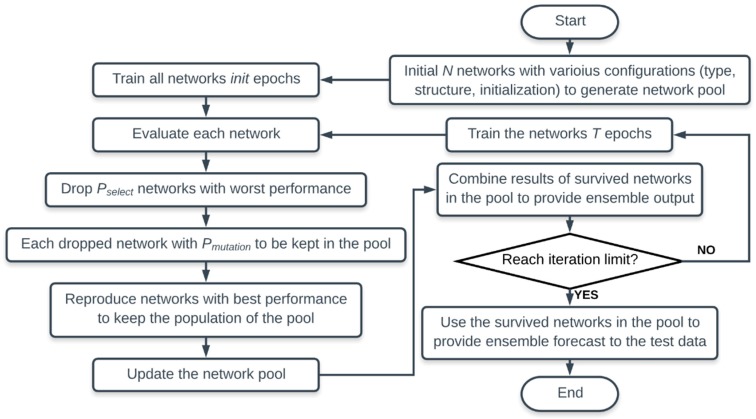
Workflow of the evolutionary ensemble method.

Workflow of EENNP is demonstrated in Figure 3. The entire process of the proposed method can be understood as an imitation of using a committee of human experts (expert system) to solve a specific problem. Each expert in the committee possesses a relatively viable neural network in his/her brain which is able to analyze similar problems. For the particular problem, each expert gives a prediction using his/her neural network in brain after a certain period of study. The predictive performance of experts is assessed every period. Members of the expert committee are updated based on the assessment results. Experts with relatively weak prediction results are invited to leave, while the voices of experts with accurate predictions rise. Then, through several rounds of member updates, members of this expert committee can be fixed to let the well- performing experts continuously contribute on the specific problem.

In addition, when implementing the proposed method, the required amount of computation of each individual as well as the entire pool can be managed. By respectively restricting the configurations (e.g., the number of layers and number of neurons/units in each layer) of networks with different types and structures, both the minimum and maximum required amount of computation of an individual in the pool is able to be set. Accordingly, the overall amount of computation of the entire pool is capable to be governed. This feature is beneficial for using this method in the HEMS described in this paper. The hardware and software of the HEMS can be designed based on bounded requirements on the amount of computation and operation time.

Furthermore, along with the dropping of weak-performing individuals, the required amount of computation of the pool in each iteration step decreases, due to the reproduced individuals are not necessary to be trained with the same data more than once.

## 4. Experimental Study

In this section, the investigated smart sensor data in the experimental study are described firstly. Subsequently, data cleansing is accomplished through a data profile analysis. Missing data and duplicates are identified and processed. Afterwards, the input data for each performance test scenario are interpreted before the evaluation criterion is provided.

### 4.1. Data Description

In this paper, the proposed EENNP method is validated on household power demand prediction with different applicational scenarios. The utilized dataset is the real time power readings of a set of anonymous western Norwegian domestic households. The power usage of each household is monitored in real time with 10.0 s interval by the smart power meters arranged in the households locally. It is assumed that in this study the smart meter monitoring power demand of each household is ideal, which means that no measurement error exists. A real time power reading is directly submitted to the remote cloud server and stored in the cloud with the data receiving time. The available data for all the households are stored in Python pickle files by day. Approximately 4 months data are available for each household. Every data row consists of three features, which are the anonymous household ID, timestamp in millisecond and power reading in Watt. The data rows with same house ID are combined and sorted by timestamp to get the dataset for each household.

From observation, it is found that the intervals between adjacent rows are not fixed, despite the sampling interval in a household is 10.0 s. Through analysis, 95.0% of available data rows are with intervals in the range [9.9, 10.1]. However, considering the total amount of data rows among the dataset of each household is at $10^{6}$ level, it is necessary to provide some attention on the rows with other intervals. Both duplicates and missing data could be unfavorable for real time/near real time prediction as well as historical analysis. Hence, in order to have a better learning of the data through machine learning, data cleansing is required to be done, which is illustrated in the next subsection.

### 4.2. Data Cleansing

Since the utilized data for each household are time series data, it is worth to keep the coherence of the data sequence on model training, rather than simply remove the portions containing duplicates and missing data to train model by the remaining data. In the dataset of a household, timestamp can be deemed as the key. The intervals between adjacent rows are analyzed to define the missing and duplicate data.

**Figure 4:**
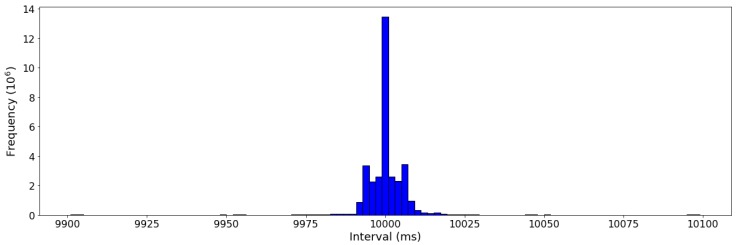
The distribution of the interval between adjacent rows ([9.9, 10.1]).

The distribution of the interval between adjacent rows among all datasets is illustrated in Figure 4. It is interesting to observe that the distribution exhibits an approximate axisymmetric along 10.0 s. A feasible reason could be that a possible origin of data interval floating comes from the communication process between the smart sensor and remote cloud. For instance, when a package with the first power reading is transmitted and received without any delay, the next package with the second power reading monitored exactly 10.0 s later is delayed 10 ms, and the third package with the following power reading gets no delay again, then the intervals between the three records should be axisymmetric along 10.0 s, which are 10,010 ms and 9990 ms, respectively.

Additionally, it is observed that all row pairs with interval in range [0, 5.0) own the same power readings within the pair. Hence, it is assumed here that if the interval between two adjacent rows is less than 5.0 s, the latter row is a duplicate. Besides, if the interval is greater than or equal to 15.0 s, it is supposed that one or multiple rows are missed between the adjacent rows, as illustrated in (2).

(2) $$\left\{ \begin{array}{cc} 0\leq \mathrm{interval}<5 & \mathrm{The}\;\mathrm{latter}\;\mathrm{is}\;a\;\mathrm{duplicate}\\ 5\leq \mathrm{interval}<15 & \mathrm{Correct}\;\mathrm{data}\\ \mathrm{interval}\geq 15 & \mathrm{Data}\;\mathrm{missing}\;\mathrm{between}\;\mathrm{the}\;\mathrm{rows} \end{array}.\right.$$

In this research, the duplicates are removed from the datasets to eliminate rows with too short intervals. Although the deletion of data row leads to loss of information, it is considered that the effect of deletion of the duplicates is relatively limited since the duplicates are with the same power readings as their previous rows. Moreover, it is required to restrict the interval between rows within a certain boundary to minimize that the impact of the interval of the time series input on the model learning.

About missing data, a rough distribution of the intervals between the rows with missing data is presented in Table 1. It is observed that the majority of the rows with missing data hold intervals within [15,60) and [120,180) seconds. The possible origins include communication errors (such as package loss, network reconnection), smart meter issues, cloud server unstable, etc.

**Figure 5:**
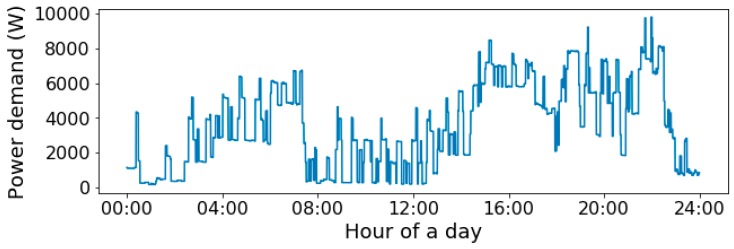
Power demand of a day.

The power readings of a day without missing and duplicate data are illustrated in Figure 5 as an example. It is observed that the power readings always remain relatively stable for certain periods. Besides, according to a brief investigated, the power reading difference between the start- and end-row of missing data is normally less than 10 W. Hence, to refill the missing data, a number of rows are evenly inserted on time between the rows with missing data until the interval is less than 15.0 s. The power readings of the inserted rows are assigned as an arithmetic progression between the start- and end-row power readings. Another missing data refilling method is discussed based on EENNP as a potential application scenario of the method in Section 5.

### 4.3. Input Data

To investigate the performance of the proposed method in the three application scenarios, single time series input, the power reading of a period (10-s), is employed to train each network individual in the pool to forecast the power demand of the next period (10-s). In the utilization in HEMS, each real-time power reading of the household is used as an input of the trained model and a forecast of the power demand of the coming 10 s is provided as the output of the model at each period.

The impacts of other inputs such as time labels (e.g., day of week, time of day) and external parameters (e.g., temperature, wind speed) on the performance of the proposed method will be discussed in our future studies.

To discuss the power demand of the coming 10 s, an assumption should be provided that the power demand among each monitoring interval is steady. Hence, the available data could represent the power demand of a specific household within the coming 10 s.

In the initialization step, an initial-training dataset is utilized to train the randomly generated network individuals in the pool. Then, in each iteration step, a new set of data is assigned towards the training set. New validation and evaluation datasets are allocated. After each iteration step, the utilized data are added towards the training set to avoid the happening of data reuse in future evaluation operations. When the evolutionary process is finished, a test set is set to investigate the performance of the model.

The performance of the proposed method at three application scenarios is investigated and discussed in the next section, which are optimizing potential network configuration set, forecasting single household demand, and refilling missing data respectively. The cleansed data without duplicates and missing rows are utilized in the training and evaluation dataset of all the three scenarios, as well as in the test set of the configuration set optimization and single household prediction. In the experiments of missing data refilling, several sets of raw data without duplicate and missing rows are randomly picked among the period of the test set. These sets are utilized on model reheating and testing. More detail on the three application scenarios are presented in the corresponding subsections in the next section.

### 4.4. Evaluation Criterion

Mean absolutely percentage error (MAPE) is adopted to evaluate all the scenarios of the discussed predictions. MAPE measures the average absolute of occurred errors as illustrated in (3).

(3) $$\mathrm{MAPE}=\sum_{j=1}^{M}\left|\frac{{\mathrm{OUT}}_{j}-{\mathrm{TGT}}_{j}}{{\mathrm{TGT}}_{j}}\right|,$$

where M expresses the total number of rows in the set to evaluate.

## 5. Performance Analyses and Discussions

In this section, the performance of three application scenarios of the proposed method on household power demand prediction is analyzed and discussed. The utilized input data are pre-processed as described in Section 4. The codes used in this study are programmed in Python 3.0 [34]. TensorFlow [35] library is adopted to implement the neural networks including configuration, initialization, training and evaluation.

### 5.1. Optimizing Potential Network Configuration Set Using EENNP

To implement the proposed method, a set of potential network configurations based on a former research should be built, while EENNP itself can be utilized on the building of potential network configuration set for a specific problem as well.

A potential configuration set includes network types, structures, and other configurations (such as activation functions of the neurons) that can form a network fit the problem properly. As discussed in Section 2. DNN and RNN with LSTM or GRU units are the three candidates on the network types. However, for the specific case regarding household power demand prediction, it is hard to clearly state which network type and structure is undoubted outstanding. Therefore, EENNP can be adopted to select the potential network configuration set.

In order to roughly control the amount of computation for each individual network, the upper limit of the number of weight vectors in an individual is specified. Choosing the number of weight vectors to represent the amount of computation of a network is because both feed-forward and learning process require to operate all the weight vectors in the network. Hence the number of weight vectors is used in the study to roughly count the amount of computation required by a network.

In a DNN, weight vectors exist in each neuron of the network to calculate the weighted summary with the input of the neuron. The number of weight vectors in the network equals the total number of neurons in the network. In an LSTM unit or a GRU, weight vectors emerge together with the activation functions which compute input. An LSTM unit exists 4 weight vectors, and a GRU unit has 3 weight vectors.

Thereupon, based on these relative relationships and considering the time of operating experiments, in this paper, the maximum number of neurons for a DNN individual is set as 384; in an LSTM based RNN, the upper limit of LSTM units can be configured is set as 96; in a GRU base RNN, the number of GRU can be set is equal to or less than 128. All the three types of the networks can be configured with 1 to 10 hidden layers. In RNNs, the activation functions in the units are given by the unit definitions. In a DNN, the activation function in the neurons can be configured from a set of activation functions. In this research, the activation function is configured by layer. Either sigmoid function or hyperbolic tangent function is able to be set as the activation function of all the neurons in a layer.

**Figure 6:**
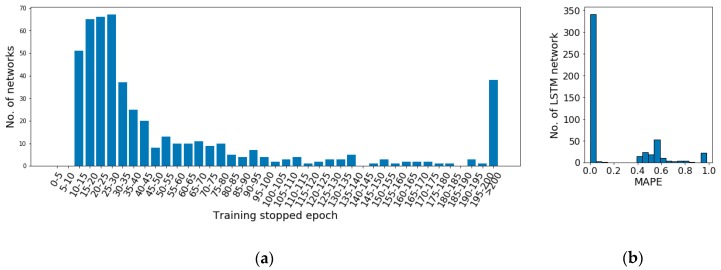
The performance of 500 random initialized networks which are randomly generated using the potential network configuration set: (**a**) Distribution of training stopping epochs; (**b**) Distribution of performance of the networks.

As an example, to illustrate the general performance of the networks in the potential configuration set, 500 random generated networks based on the set are initialized randomly and trained using the same cost function and learning algorithm, which are mean squared error and Adam optimization algorithm respectively. The data of a random household are used as input of the networks. Each network is trained 200 epochs unless early-stopping is triggered. The distribution of training stopping epoch as well as the distribution of performance of the networks are illustrated in Figure 6.

It can be observed in Figure 6a that after 10–15 epochs of training, the network stopping epochs widely distribute among the epoch ranges. More than 60% networks stop within 40 epochs. However, the rest of the networks stop gradually along with the training. Even there are 7.6% of the networks which do not trigger early-stopping after 200 epochs of training. Moreover, the performance of the networks on the test set illustrated in Figure 6b shows that the majority of the networks achieve significantly better performance (MAPE < 0.1) than others. Therefore, an analysis on the potential configuration set is required to remove network configurations normally with weak performance and with too long learning period to improve the model performance. It is required to notice here that network initialization is an important factor on the network performance. It is always possible to achieve a worse random initialization causing a slow or weak learning. The target of this analysis is to remove the commonly emerging weak configurations to improve the overall performance of the pool.

An example of manually optimizing the potential network configuration set is provided as follow:

Through quantitative analysis, for training performance, in the 100 networks with better performance (MAPE < 0.0012), 5% of them are DNNs, LSTM based RNN accounts 49%, GRU based RNN occupies 46%. All the better performing RNNs are with 1–4 layers. It is observed that LSTM and GRU are relatively more suitable for the study on household power demand prediction.

In addition, as shown in Figure 6b, among all the networks, 31.2% networks achieve MAPE on test set higher than 0.1 (10%). Among them, 86.5% are DNNs, 7.1% are GRU based RNNs, 6.4% are LSTM based RNNs. Within the LSTM and GRU based networks with MAPE higher than 0.1, there are 90.0% and 100% of them with MAPE greater than 0.9 respectively. With a closer observation, all of weak performing RNNs with greater than 0.4 MAPE hold more than 4 layers, while their stopping epochs are with no significant particularity comparing with other networks.

Moreover, from the perspective of stopping epoch, 25.4% of networks with longer than 40 epochs training are DNNs, 46.0% are LSTM based RNNs, 28.5% are GRU based RNNs. It is not able to observe that the training period of DNNs hold a significant advantage comparing with RNNs.

To conclude, LSTM and GRU based RNNs with their outstanding performance are considered to suit the prediction of the experimental study case, household power demand prediction. The preferred network structures achieved by enumerative based quantitative analysis, are RNNs with 1–4 layers, which is set as the optimized potential network configuration set.

As described above, the entire process of optimizing potential network configuration set could spend a considerable period which has to be done manually. It is hard to be implemented in a HEMS or other distributed scenarios, since it is too costly to provide manual selecting/turning for each distributed located neural network prediction. Therefore, the proposed EENNP method is utilized to realize the optimizing process automatically.

As an operating example suitable for the data we have as well as our experimental equipment, the total number of network individuals in the pool is configured as N=100. The evolutionary parameters are set as $init=20,\;T=5,\;P_{select}=10\%,\;P_{mutation}=5\%$, and the iteration limit is set as 50 epochs. Comprehensive considering the data we have and the requirement of evolutionary process, 30% of the time series data at beginning is set to the initialization step. Successively, each iteration uses the subsequent 10% data for further process, among them 9% are for training and 1% are for validation and evaluation. The validation and evaluation of the networks use a same dataset. Then, the next 10% of the data are utilized for testing. Within the evolutionary training, 78 among the 100 networks are dropped. The surviving ones with their types and number of layers are illustrated in Table 2, where 3.1% are DNNs, 37.5% are LSTM based RNNs, 59.4% are GRU based RNNs. Thereinto, the 93.5% of RNNs are with 1–4 layers. Therefore, 1–4 layers RNNs are selected as the optimized potential network configuration set for the training of the other application scenarios.

It can be observed that the selection result achieved by using EENNP is very similar with the manual analysis result, but with much less manual operating time.

### 5.2. Power Demand Prediction Using EENNP

Adopting the optimized potential network configuration set achieved in the last subsection, the performance of EENNP on specific households is discussed in this subsection. The impact of picking different evolutionary parameters on the model performance is investigated as well. Input data preparation process is mentioned in Section 4.

**Figure 7:**
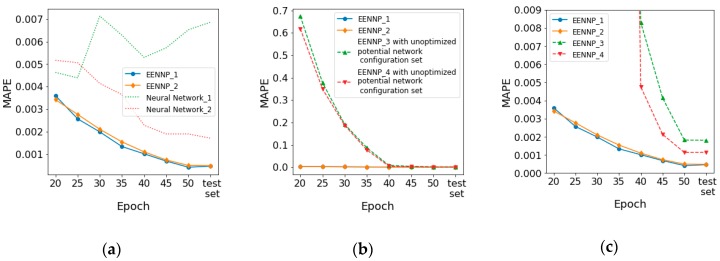
The performance of EENNP methods using different potential network configuration set: (**a**) The performance of two runs of EENNPs with the optimized potential network configuration set and two networks within the final survival population; (**b**) The performance of four runs of EENNPs in which two uses the optimized potential network configuration set and the other two uses the unoptimized set; (**c**) The comparation between the four EENNPs in detail.

As an operating example suitable for the data we have as well as our experimental equipment, the total number of network individuals in the pool is configured as N=50. The evolutionary parameters are set as $init=20,\;T=5,\;P_{select}=10\%,\;P_{mutation}=5\%$, and the iteration limit is set as 50 epochs. Same training, evaluation and test datasets are determined as in the scenario of optimizing potential network configuration set. The proposed method is utilized to train the available data of a household 2 times. The performance of the two experiments are illustrated as EENNP_1 and EENNP_2 in Figure 7a by solid lines. The performance of two predictors are introduced here as comparisons. The MAPE of a naïve predictor, which uses the power reading now to forecast the next, is 0.0868. A simple 1-layer, 1-neuron ANN model, which is trained by all the training data 50 epochs and then evaluated by the test dataset, has MAPE = 0.0589. It is observed that the proposed method achieves better performance comparing with naïve or simple predictors.

Two neural networks within the final survival population in the pool are shown in Figure 7a as Neural Network_1 and Neural Network_2 using dotted lines. It is observed that comparing with neural network individuals within the final survival population, the proposed method usually can achieve a better performance. In addition, comparing with the unexpectable performance achieved by using a network individual to forecast power demand, the results obtained through the proposed ensemble method demonstrate a relatively better stability. The performance of EENNP using the unoptimized potential network configuration set (1–10 layers DNN/RNN) on the same data with the same evolutionary parameters are tested 2 times. The prediction results of the test set are illustrated in Figure 7b,c as EENNP_3 and EENNP_4 by dash lines. The performance of EENNP_3, EENNP_4 indicates a similar stability as EENNP_1 and EENNP_2. A possible source of the stability could come from the potential network configuration set. The possibility of obtaining a proper prediction within a set of configurations could be relatively steady. With the same evolutionary parameters, the better performing networks are gradually selected out through iteration steps.

Based on this reason, although it could happen that within a population of networks do not exist any with a proper combination of configuration and initialization, the possibility of obtaining a better prediction by using EENNP is significantly higher that using one neural network with a randomly chosen type and structure. Moreover, without manually tuning a network initialized by random numbers, EENNP provides an automatic method to achieve a better prediction.

In addition, the comparison between curves of EENNP_1&2 and EENNP_3&4 (in Figure 7b,c) illustrates the difference on performance using different potential network configuration sets. By using the optimized configuration set, the pools perform significantly better than the pools using the unoptimized set at the beginning iteration steps. Their performance becomes closer at the last steps. This phenomenon is reasonable since within the bigger but unoptimized set of potential configurations, the possibility of achieving an individual which is generated and initialized properly is lower than within a smaller, optimized set. Therefore, more weak performing individuals are generated in EENNP_3&4, which requires more iteration steps to be removed. With better performing individuals generated in EENNP_1&2, the possibility of achieving even more excelling performing individuals is higher. In addition, with better performing individuals in the pool, it is reasonable to achieve better ensemble results through tradeoff among the survival individuals.

Moreover, it is able to be observed in Figure 7a,c that the performance of test set could be worse than the learning period. It is because that the test data are always brand-new data rows for the models, which could lead unstable on model performance. The decline of performance could cause by the rising of new behavior pattern in the data, model over-fitting, etc. One reason of new behavior pattern rising could be that the available data are only for 4 months, new power demand patterns maybe arise when a new season comes. About over-fitting, it can happen on any neural network-based models (as shown in Figure 7a: Neural network_1), even though new evaluation data are kept introduced into the input dataset. Among the tested EENNPs, this phenomenon is not particularly serious.

**Figure 8:**
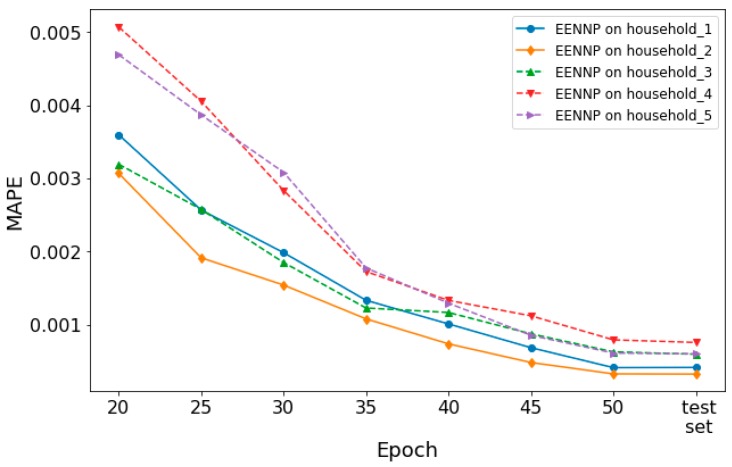
Performance of EENNP on different households.

Additionally, the performance of the optimized potential configuration set based EENNPs on different households is tested. In Figure 8, the performance of EENNP on 5 different households is illustrated. It is observed that the performance curves of the networks are with a similar trend. If we look into the details of their performance, the 5 EENNPs can be divided into two groups. The performance on household 4&5 is weaker than on household 1&2&3 at beginning. Then during the iteration steps, household 4&5 catches up the others. This phenomenon is similar with the comparison between EENNPs using optimized and unoptimized configuration sets in Figure 7b,c (EENNP_1&2 and EENNP_3&4), but with less performance gaps. Hence it is deduced that the cause of having this phenomenon could be that the households have different patterns of power demand individualities, which can be influenced by the number of persons living in the household, their ages, habits, etc. For instance, the power demand pattern of young couples with two children could be very different with single retired persons. It is possible to have more specific potential network configuration sets for households with different patterns. However, it is not the main focus of this paper. The corresponding research will be conducted in our future studies.

**Figure 9:**
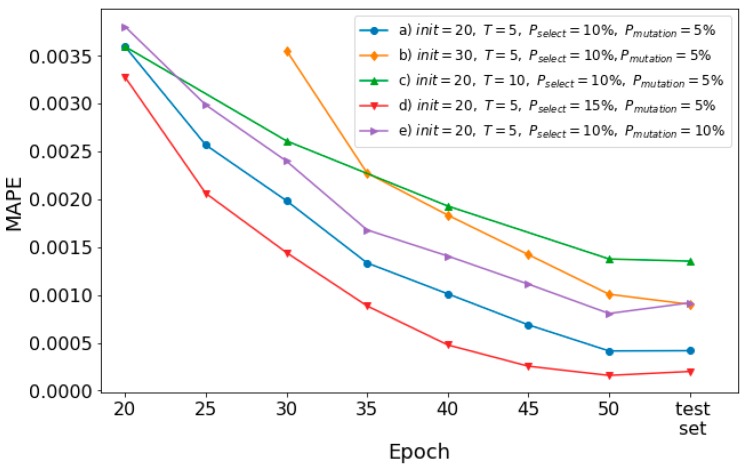
The impact of evolutionary parameters on model performance.

In the following, the impact of evolutionary parameters on the model performance is investigated. Using the optimized configuration set, 5 EENNPs are tested using the data of a household with different evolutionary parameter sets, which are: a) $init=20,\;T=5,\;P_{select}=10\%,\;P_{mutation}=5\%$; b) $init=30,\;T=5,\;P_{select}=10\%,\;P_{mutation}=5\%$; c) $init=20,\;T=10,\;P_{select}=10\%,\;P_{mutation}=5\%$; d) $init=20,\;T=5,\;P_{select}=15\%,\;P_{mutation}=5\%$; e) $init=20,\;T=5,\;P_{select}=10\%,\;P_{mutation}=10\%$. To keep the pace of learning among the experiments with different evolutionary parameter sets, the set b) uses the first 50% of available data for the initialization step. In each iteration step in the experiment on set c), 20% of data are used. The performance of the models is demonstrated in Figure 9.

Comparison between a) (the blue line with circle markers) and b) (the yellow line with diamond markers) illustrates that with a longer initialization step, the model performance is getting weak. It is because a) obtains 2 more iteration steps than b), within which 10 weak performing individuals are dropped from the pool and 10 better performing individuals are reproduced (if no mutation happens). It could cause a difference on model final performance. In addition, with 10 more epochs of initialization without dropping, the amount of computation utilized by b) is more than a), which leads b) as a worse evolutionary parameter set. However, depending on different research questions, the suitable ini could be various. A very less init could also cause that potentially better performing individuals are dropped at early steps.

If we compare a) and c) (the green line with triangle_up markers), it is observed that with more epochs of training on each iteration step, the performance of the model declines. It is reasonable since a) achieves 2 more rounds of selections with the same amount of computation. However, similar as for init, a too short training time could also not good for the individuals to learn the environment.

The impact of increasing dropping and reproducing rate is demonstrated by a) and d) (the red line with triangle_down markers) in Figure 9. With a greater rate of selection, a better model performance is achieved. Based on our quantitative analysis illustrated in Figure 6a, where the majority of networks achieve their first early-stopping before epoch-40. It means the individuals with proper combinations of configuration and initialization are most likely able to show out their fitness before epoch-40. Therefore, the iteration steps could easily select these individuals out, to lead a better performing model than using d). However, for situations that the potential better performing ones cannot show out their fitness at beginning, a greater $P_{select}$ could lead a reduction on model performance. Moreover, a greater $P_{select}$ may also lead the diversity of the pool decreasing. If the number of individuals of one combination of configuration and initialization is too large in the entire population, it could also weaken the stability of the prediction performance. The purple line with triangle_right markers illustrates the model performance using e). Comparing e) and a), it is observed that with a higher $P_{mutation}$, the model performance decreases. It is because that a higher $P_{mutation}$ could cause weak performing individuals are remained in the pool through the iteration steps. However, for situations that potential proper networks do not clearly emerge at early steps, a certain rate of mutation could be useful to keep the potential networks within the pools.

### 5.3. Missing Data Refilling using EENNP

Data correctness is the basis of a good prediction. In order to handle the missing data in the achieved raw datasets in a more reliable way to imitate the power demand during the missing period, the use of EENNP is discussed in this subsection to generate refilling rows for the missing power demands.

A potential use case of the missing data refilling is at the cloud server part. Some real-time analyses could be operating on the cloud which require the real-time power readings as an input. Data missing in this situation could influence the real-time analyses. At the same time, as a rare happening event, data missing is not necessary for the cloud server to have an always-on refilling method to handle. Therefore, a lightweight mechanism is required to be triggered whenever missing data happens to generate a relatively reliable substitute for the missing power reading.

Based on the requirements discussed above, the refilling method we used in data preprocessing (Section 4.2) is no longer available, since we cannot obtain the required information on real-time. For instance, when the next data reading will be received by the cloud server. Therefore, a missing data refilling method using EENNP method is developed here: A pool with well-performing individuals for a specific household is achieved beforehand by using EENNP method, as described in Section 5.2. Then the pool is frozen there until a data missing is detected. A total of 20 min of historical data before the missing point (about 120 rows) are used to reheat the pool T epochs before a forecast of the missing data is given.

The household utilized to train the EENNP illustrated in Figure 7 is employed to build a test. The pool is initialized and evolutionarily trained as described in Section 5.2. For the testing set, 5 portions of raw data without duplicate and missing rows are randomly picked from the test period (last 10% of available data) to organize the missing rows as well as the corresponding reheating sets, as described in Section 4.3. EENNP_1 illustrated in Figure 7 with $init=20,\;T=5,\;P_{select}=10\%,\;P_{mutation}=5\%$ is utilized to execute the experiments. The test results show that with reheating, the performance of missing data refilling is similar with prediction. The average MAPE of the 5 tests is 0.0005, which is significantly better than the naive predictor, which is another missing data refilling method capable for (near) real-time treatment.

## 6. Conclusions

In this paper, based on the requirements of power demand prediction on establishing HEMS, a novel EENNP method is proposed to achieve a population of well-performing networks with proper combinations of configuration and initialization through evolutionary method automatically. The survival networks are combined to obtain the power demand prediction by ensemble method.

In the experimental study, the power reading data monitored in multiple households are cleansed based on data profile analysis. The processed data are utilized on three application scenarios. The first application scenario is to optimize the potential network configuration set. The optimized potential network configuration set using EENNP achieves a similar result to manual optimization. The second application scenario is to forecast the power demand of a single household. The evaluation of prediction results shows that the proposed EENNP method performs significantly better than the naive and simple predictors. And at the same time the ensemble prediction performs certain stability comparing with single network within the final survival population of the pool. Additionally, impacts of evolutionary parameters on model performance are investigated. The third application scenario is to refill the missing data for the use of (near) real-time analyses. It is observed that the refilling results by using EENNP based method are better than naive predictor.

The limitations of this study include: Most of the evolutionary parameter values and datasets (training, testing, validation, etc.) utilized in the study are decided based on empirical experimental results. No theoretical determination regulation is practically complied. It is because that the determination of evolutionary parameters and datasets could relate to the realization situation of the method in HEMS, which is not the focus of this paper. In addition, more inputs such as energy appliances’ real-time status, temperature, etc. could be introduced into the model training to advance the model performance. Moreover, different patterns of power demand individuality appearing in different household could also be explored in more detail. The limitations of this paper discussed above would be our further topics to discuss.

## Figures and Tables

**Table 1:** A rough interval distribution of the rows with missing data.

Interval (s)	Proportion (%)
[15,60)	45.8
[60,120)	4.2
[120,180)	44.8
>180	5.2

**Table 2:** Number of layers of the survival network.

Network Type	1-Layer	2-Layer	3-Layer	4-Layer	5-Layer	6-Layer	7-Layer	8-Layer	9-Layer	10-Layer
DNN	0	0	1	0	0	0	0	0	0	0
LSTM based RNN	3	3	4	2	0	0	0	0	0	0
GRU based RNN	8	4	1	4	2	0	0	0	0	0
